# Impact of child’s migration on health status and health care utilization of older parents with chronic diseases left behind in China

**DOI:** 10.1186/s12889-021-11927-x

**Published:** 2021-10-19

**Authors:** Yuxin Liu, Jia Wang, Ziqi Yan, Rui Huang, Yan Cao, Hongxun Song, Da Feng

**Affiliations:** 1 School of Medicine and Health Management, Tongji Medical College, Huazhong University of Science and Technology, Wuhan, 430030 Hubei China; 2 School of Pharmacy, Tongji Medical College, Huazhong University of Science and Technology, Wuhan, 430030 Hubei China; 3 School of Political Science and Public Administration, Wuhan University, Wuhan, 430072 Hubei China

**Keywords:** Health status, Health care utilization, Chronic diseases, Older parents, Left behind

## Abstract

**Background:**

Adult child are used to taking the responsibility of taking care of their older parents in Chinese culture. However, the migration of adult child is not uncommon now in the context of urbanization in China. The purpose of this study is to explore the impact of child’s migration on health status and health care utilization of older parents with chronic diseases left behind.

**Methods:**

The data of the 2015 nationally representative longitudinal survey of the aged population in China were used in this study. Binary logistic regression was used to evaluate the impact of adult child’s migration on health status and health care utilization of older parents with chronic diseases left behind.

**Results:**

About a quarter of the respondents (25.5%) had at least one migrant child. Most of the respondents (86.6%) rated their health as poor, and 42.0% of them suffered from physical limitations. Nearly half of the respondents (45.0%) had depressive symptoms, but the vast majority (88.2%) were generally satisfied with their lives. Only a quarter of the respondents received outpatient treatment in the past month while only one fifth of them received inpatient visits in the past year. After controlling for other demographic and socioeconomic variables, it was found in this study that those who with migrant child were more likely to report poor self-rated health (OR = 1.26; 95% CI 1.01–1.58), not satisfied with general life (OR = 1.28; 95% CI 1.03–1.59) and seek outpatient visits (OR = 1.22; 95% CI 1.03–1.43) than those who without migrant child.

**Conclusion:**

Our study found that there is a negative association between migration of adult child and physical health, mental health and health care utilization of older parents with chronic diseases left behind, which means a comprehensive effect on their health status. Further health policies should focus on improving the well-being of older parents with chronic diseases left behind.

## Background

Chronic non-communicable disease is a general term for a class of diseases with hidden onset, long incubation period, long and slow course, unsustainable condition, no clear indication of “curing”, including cardiovascular diseases, diabetes, cancer, chronic respiratory diseases and others [1], which are common in the aged population worldwide today [2]. On the other hand, China has the largest aged population in the world and it has been ageing continuously [3]. There were 14.4 million older people in China in 2015, which equates to 10.5% of the total population, and this percentage is expected to increase to 34.1% by mid-century [4]. Along with this demographic transformation, there is a health transition from communicable diseases to chronic non-communicable diseases [5]. China is now facing the burden of accelerating population ageing and increasing numbers of patients with chronic diseases, along with the lack of the institutional support to meet the needs of the older patients with chronic diseases. “Medium and Long-Term Planning for the Prevention and Treatment of Chronic Diseases in China (2017-2025)” shows that the number of people dying of chronic diseases in China accounts for 86% of the total deaths, and the disease burden caused by chronic diseases accounts for more than 70% of the total disease burden, which has seriously affected the health of Chinese residents.

To respect and support one’s parents has been emphasized in Chinese culture [6], the aged population in China traditionally relies on their children to provide personal care and economic support [7, 8]. According to extant reports, 88.7% of the aged need the help of family members in their daily lives [9]. However, due to the large-scale internal migration under China’s rapid growth of economic and process of urbanization, the traditional system of family support has been profoundly changed. The home-based care for the aged faces challenges, especially when their children are migrants [10]. Given the long distance of migration, they cannot provide daily care for their parents left behind, who may have problems of self-care. It has been reported that there were about 277.5 million rural laborers working in urban areas in 2015, accounting for 36% of the total 770 million laborers in China [5]. The migration of labor force has led to a decreasing numbers of children available to take care of their older parents, which eroded the foundation of traditional family support relationships [11–13] and raised concerns about whether the absence of adult child in a household will affect health status and health care utilization of older parents left behind, especially in the absence of a mature social security system for the aged, including public pensions, health insurance and social care [9].

The issues of public health, economy and migration are generally intertwined [14–16]. The relationship between one’s migration status and health status of left-behind parents is an emerging field of study and existing researches reflect different findings [17–19]. Intuition suggests that one’s migration may lead to the deterioration of the emotional and physical conditions of older parents left behind, affecting their health in the long run [20]. Relevant research notes that young adult’s migration has severe negative impact on their older parents, that is, loneliness, isolation and loss of economic support [21]. A study on Mexico-US immigrants found that there was a causal relationship between older parents’ poor health outcomes and their children’s migration to the United States [22]. Besides, similar negative effects of one’s migration on left-behind parents’ health have also been found in India [23]. Evidence from rural China also indicated that one’s migration status significantly lowers parents’ overall health outcomes [24]. However, on the other hand, evidence from the literature also reflects the economic gains of migration, showing that migrants can positively affect the health of those left behind through economic support [12, 25, 26]. Migrant children are generally able to earn more in other regions, so their older parents left behind can have easier access to medical services through remittances from their migrant adult children, which means migrants can bring family members positive health outcomes by increasing household income [17]. Besides, it was also found that children’s migration was associated with lower levels of parents’ depression [27].

Although the migration of adult child may have serious implications on health status and health care utilization of older parents left behind, the possible health effects on older parents with chronic diseases left behind in China are still unclear. The aim of this study is to explore the effect of adult child’s migration on health status and health care utilization of older parents with chronic diseases left behind in the context of the urbanization in China [28]. In-depth research on these issues will provide the government with the information needed for policy-making. The results of this study will help to fill gaps in related research and provide understandings to the design of appropriate interventions to the internal migration in China.

## Methods

### Sources of data and study design

The data used in this study came from the China Health and Retirement Longitudinal Study (CHARLS) which targeting the middle-aged and elderly population in China. CHARLS is a nationally representative longitudinal survey of people aged 45 years and above conducted by the Institute of Social Science Survey, Peking University [29]. CHARLS contains a wide range of information including demographic, family, health status and function, health care and insurance, work information, income and expenditure and so on. The survey of CHARLS selected 450 villages or communities in 150 counties of 28 provinces of China using Probability Proportionate to Size Sampling [30], which represents China to a large extent. All data in CHARLS were collected by well-trained interviewers using structured questionnaire through face-to-face, computer-assisted personal interviews and can be obtained on the corresponding website. The original CHARLS was approved by the Ethical Review Committee at Peking University (IRB00001052–11015). All participants provided signed informed consent before the data was collected.

In this study, we used data from the CHARLS 2015 only, which was conducted from July to August 2015. Since CHARLS is a longitudinal survey and the respondents are followed up every three years, the answer to some questions is “No change”, we matched the individuals of CHARLS 2015 and CHARLS 2013 and CHARLS 2011 based on their individual ID to collect the specific answer we need. To find the association between adult child’s migration and health status and health care utilization of their older parents with chronic diseases left behind, we narrowed the sample to respondents who reported chronic diseases in 2015 with at least one child and provided complete data in all the surveys. As a result, a total of 3919 respondents were included in this study.

### Measurement

#### Dependent variables

The WHO defines health status as a Comprehensive indicator includes physical, mental and social well-being instead of the absence of disease or frailty [31]. The CHARLS data used in this study provide multiple individual-level health indicators to analyze the health status, including self-rated health (SRH), activities of daily living (ADL), instrumental activities of daily living (IADL), self-rated life satisfaction (SRLS) and the 10-item Center for Epidemiologic Studies Depression Scale (CES-D-10). These variables have also been widely used in other researches on the impact of children’s migration on health status of the aged [32]. Since health is a multi-dimensional concept, we included physical health, mental health and health care utilization of older parents with chronic diseases left behind as health outcomes in this study.

##### Physical health

Physical health were measured by two indicators: self-rated health (SRH) and physical limitations. SRH represents the own assessment of participants. CHARLS provides two dimensions of self-assessment health status, respondents were divided into two groups randomly, rating their health status from excellent to very good, good, fair, poor and from very good to good, fair, poor, very poor. In this study, SRH were coded as a dichotomous indicator, we considered SRH of good or above in both dimensions as the cutoff of good self-perceived health [33]. For regression model applicability, we redefined “excellent”, “very good” and “good” as good health status assigned a value of 1 and “fair”, “poor” and “very poor” as bad health status assigned a value of 0. The CHARLS questionnaire asked the respondents to complete two self-health assessments at the beginning and the end of the interview respectively. Considering that some of the respondents may not be very clear about their health status at the beginning of the interview, the second answer was chosen in this study because of its higher credibility, and for respondents who did not have a second answer, we chose the first answer. The variable physical limitations were also chosen to represent physical health because such a method allowed us to gauge older adults’ difficulties in activities of daily living. Disability was measured by two scales: the 6-item scale of activities of daily living (ADL) refers to physical activities such as dressing, bathing and eating and the other 5-item scale of instrumental activities of daily living (IADL) refers to self-care ability of life such as preparing meals, shopping and taking medications [34]. The options were set to “No, I don’t have any difficulty”, “I have difficulty but can still do it”, “Yes, I have difficulty and need help”, “I cannot do it”, and corresponding score was “0 points”, “1 point”, “2 points”, “3 points”. Having no disability was defined as having no difficulty in all ADL and IADL items which means the sum of the responses of all the 11 questions equals 0 [34].

##### Mental health

Mental health were also measured by two indicators: self-rated life satisfaction (SRLS) and the 10-item Center for Epidemiologic Studies Depression Scale (CES-D-10) questionnaire. To assess the SRLS, respondents were asked to select from “Completely satisfied”, “Very satisfied”, “Somewhat satisfied”, “Not very satisfied” and “Not at all satisfied” to answer the question of how they think about their life-as-a-whole and how they satisfied with it in CHARLS. In this study, the first three items were defined as “Satisfied” and the last two were defined as “Not satisfied”, assigned 1 and 0 respectively. Depression scores were calculated according to the CES-D-10 which includes 10 questions. The response for each CES-D-10 question was on a four-scale metric: rarely, in some days (1–2 days per week), occasionally (3–4 days per week), and most of the time (5–7 days per week). Participants’ responses were recoded from 0 (rarely) to 3 (most of the time) for the negative questions and from 3 (rarely) to 0 (most of the time) for the positive questions (The item “I felt hopeful about the future” and “I was happy”). We added up the scores to create a depression indicator ranging from 0 to 30, and higher values suggest higher levels of depressive symptoms. The CES-D-10 indicates good validity and reliability, with a Cronbach’s alpha of 0.815 [35]. A cut-off score of ten or higher was adopted to indicate the respondents who had significant depressive symptoms in this study [36].

##### Health care utilization

Health care utilization was measured based on the following questions: The binary variable of the outpatient visits in the last month was measured by the question “Have you visited a public or private hospital, public health center or clinic, health worker’s or doctor’s practice, or been visited by a health worker or doctor for outpatient care in the past month?”, respondents who did go to a health care provider were coded as 1 for utilizing outpatient care while those who did not were coded as 0 for underutilizing outpatient care; The other one is the binary variable of the inpatient visits in the last year which was measured by the question “Have you received inpatient care in the past year?”, respondents who received inpatient care were coded as 1 for utilizing inpatient care while those who did not were coded as 0 for underutilizing inpatient care. The answers to the question were “yes” or “no”.

#### Independent variables

##### Migration of adult child

Numerous variables are available in CHARLS. In this study, we focused on the chronically ill elderly people who had at least one child, using migration of child as a main independent variable. By enquiring “Where does this child normally live now?” with seven response options (This household, and economically dependent; This household, but economically independent; The same or adjacent dwelling/courtyard with me; Another household in my permanent address’s village/neighborhood; Another village/neighborhood in my permanent address’s county/city/district; Other: province_city_county/city/district, _village/neighborhood; Abroad), we collected information about their child’s habitation. In this study, having a migrant child was defined as having a child living in another county/city/district in the same province, in another province and abroad [5]. In other words, people who responses “Other: province_city_county/city/district, _village/neighborhood” and “Abroad” were considered to have a migrant child. This variable was divided into two categories: having migrant child coded as 1 and having no migrant child coded as 0.

#### Control variables

Referring to relevant literature on health status of the aged, this study adopted some other demographic and socioeconomic variables as control variables. Demographic variables included age, gender, marital status and Hukou status [37]. Socioeconomic variables included pension insurance, education and annual household spending. Age was allocated into two categories: 60 years and above and 45 to 59 years. Gender was categorized into male and female. Marital status was allocated into two categories: Married contains married, cohabitating and Single contains separated, divorced, widowed, never married. The Hukou system was established to regulate the flow of rural migrants into cities, which is a formal and legal way to bind the related welfare of urban residents [38]. Hukou is a Chinese word refers to the national household registration, all Chinese residents have a Hukou status which is generally their village or city of birth, Hukou status was categorized into Rural and Urban. Pension insurance was categorized into having or not. Education was allocated into three levels: No formal education (illiterate), Elementary school and below, Middle school and above [37]. Considering the large number of missing values and extreme values of income measure in CHARLS 2015 and the potential underestimation of income due to deliberate underreporting [39], annual household spending was used as the measure of economic status instead of income in the model. Annual household spending was categorized into three levels: low (less than 1000 CNY), moderate (1000 CNY-5000 CNY) and high (5000 CNY and more) [40], 1 USD = approx. 6.5 CNY.

### Statistical analysis

The analysis of this study was limited to those who reported at least one category of chronic disease and had at least one child. Univariate analysis and multivariate analysis were used to analyze the data in this study. We first used univariate analysis and descriptive analysis to describe the respondents’ demographic and socioeconomic characteristics. After controlling for the potential confounders, multivariate analysis in the form of logistic regression was used to assess the relationship between having migrant child and physical health, mental health, health care utilization of parents left behind. Analyses were performed using SPSS version 21.0.

## Results

### Demographic and socioeconomic characteristics of the sample population

About one-third of the respondents aged 45–59 while about two-thirds aged 60 and above, and the proportion of female respondents was slightly higher than that of male respondents. Most of the respondents were married, and there were more respondents with rural Hukou than those with urban Hukou. The large majority of respondents had no pension insurance. Nearly a quarter of the respondents had no formal education, while nearly half of the respondents had a degree in elementary school or below, and the remaining respondents (30%) had finished middle school or had a higher degree. About 20% of the respondents reported low spending capacity and 30% reported moderate, while nearly half of the respondents had a high level of annual household spending. About a quarter of the respondents had migrant child (Table 1).

**Table 1:** Selected background characteristics of the older parents with chronic diseases left behind

Characteristics		%	N
**Age group**	45–59 years	37.2	1456
	60 years or +	62.8	2463
**Gender**	Male	45.4	1780
	Female	54.6	2139
**Marital status**	Married	78.7	3083
	Single	21.3	836
**Hukou status**	Rural	77.0	3016
	Urban	23.0	903
**Having pension insurance**	No	88.0	3447
	Yes	12.0	472
**Level of education**	No formal education	23.6	925
	Elementary school and below	45.3	1774
	Middle school and above	31.1	1220
**Annual household spending**	Low (less than 1000 CNY)	18.1	708
	Moderate (1000 CNY-5000 CNY)	32.7	1282
	High (5000 CNY and more)	49.2	1929
**Migration of child**	No	74.5	2921
	Yes	25.5	998
**Total**		100.0	3919

1 USD = approx. 6.5 CNY

### Prevalence of different types of chronic diseases among the sample population

As presented in Table 2, A total of 14 types of chronic diseases were counted in CHARLS. Among all types of chronic diseases, the prevalence of arthritis or rheumatism was the highest, reaching 53.5%. In addition, prevalence of cardiovascular diseases were relatively high. For example, nearly half of the participants had hypertension and more than a quarter had heart attack. Besides, about a third of the participants suffered from stomach or other digestive disease (Table 2).

**Table 2:** Prevalence of different types of chronic diseases among the older parents left behind

Types of chronic diseases		%	N
**Hypertension**	No	55.5	2177
	Yes	44.5	1742
**Dyslipidemia**	No	78.1	3060
	Yes	21.9	859
**Diabetes or high blood sugar**	No	87.0	3409
	Yes	13.0	510
**Cancer or malignant tumor**	No	98.5	3859
	Yes	1.5	60
**Chronic lung diseases**	No	83.0	3254
	Yes	17.0	665
**Liver disease**	No	93.9	3681
	Yes	6.1	238
**Heart attack**	No	74.6	2923
	Yes	25.4	996
**Stroke**	No	96.4	3776
	Yes	3.6	143
**Kidney disease**	No	88.5	3468
	Yes	11.5	451
**Stomach or other digestive disease**	No	66.1	2589
	Yes	33.9	1330
**Emotional, nervous, or psychiatric problems**	No	98.3	3854
	Yes	1.7	65
**Memory-related disease**	No	97.2	3808
	Yes	2.8	111
**Arthritis or rheumatism**	No	46.5	1822
	Yes	53.5	2097
**Asthma**	No	92.8	3635
	Yes	7.2	284
**Total**		100.0	3919

### Health status and health care utilization

Table 3 shows that most (86.6%) of the respondents rated their health as poor, and a higher proportion of respondents who with migrant child rated a poor health compared with those who without migrant child. Furthermore, part (42.0%) of the respondents reported that they suffered from physical limitations, and a slightly higher proportion of respondents without migrant child reported suffered from physical limitations compared with those whose child had migrated was observed. On the other hand, nearly half (45.0%) of the respondents had depressive symptoms, but the vast majority (88.2%) were generally satisfied with their lives. Besides, respondents who did not have migrant child were more likely to be satisfied with their lives, but no significant difference was observed in depressive symptoms between the respondents according to the migration status of child. Moreover, only a quarter of the respondents received outpatient treatment in the past month while only one fifth of them received inpatient visits in the past year. Interestingly, there was a higher proportion of the respondents who with migrant child seeking outpatient visits compared with those whose child did not migrate, but no difference was observed among respondents in terms of inpatient visits (Table 3).

**Table 3:** Health status and health care utilization among the older parents with chronic diseases left behind according to the migration status of child

	With migrant child	Without migrant child	Total
**Self-rated health**			
Good health	11.3	14.1	13.4
Poor health	88.7	85.9	86.6
**Physical limitations**			
Yes	40.9	42.3	42.0
No	59.1	57.7	58.0
**Depressive symptoms**			
Yes	44.8	45.1	45.0
No	55.2	54.9	55.0
**Self-rated life satisfaction**			
Satisfied	86.3	88.9	88.2
Not satisfied	13.7	11.1	11.8
**Outpatient visits**			
Yes	27.4	23.8	24.7
No	72.6	76.2	75.3
**Inpatient visits**			
Yes	19.1	19.7	19.6
No	80.9	80.3	80.4
**Total**	100.0	100.0	100.0
**N**	998	2921	3919

### Multivariate analysis

In our study, binary logistic regression was used to evaluate the impact of the adult child’s migration on health status and health care utilization of older parents with chronic diseases left behind after controlling for other demographic and socioeconomic variables.

Logistic regressions were performed on 6 dependent variables separately and corresponding odds ratios were presented in Table 4. The control variables included in the models were classification indicators of age (45–59, 60 or above), sex (male, female), Hukou (rural, urban), marital status (single, married), level of education (no formal education, elementary school and below, middle school and above), pension insurance (no, yes) and annual household spending (low, moderate, high). In the regression models, the reference group of the independent variable was “no migration of adult child”.

**Table 4:** Odds ratio (OR) and 95% confidence interval (CI) for the effect of child’s migration on physical health, mental health and health care utilization of parents with chronic diseases left behind based on logistic regression

	No migration of adult child = reference category		
Dependent variables	OR	CI	Cox & Shell R square
**Physical health status (*n*** **= 3, 978)**			
**Self-rated health (ref: Good)** **Poor**	1.26*	1.01–1.58	0.009
**Symptoms of physical limitations (ref: No)** **Yes**	1.01	0.87–1.17	0.042
**Mental health status (n = 3, 978)**			
**Self-assessed general life satisfaction (ref: Satisfied)** **Not satisfied**	1.28*	1.03–1.59	0.014
**Symptoms of depressive (ref: No)** **Yes**	1.02	0.88–1.18	0.050
**Health care utilization behavior (n = 3, 978)**			
**Outpatient visits in the last month (ref: No)** **Yes**	1.22*	1.03–1.43	0.018
**Inpatient visits in the last year (ref: No)** **Yes**	1.04	0.87–1.26	0.055

*Notes: Logistic regression was run separately for each dependent variable*

*Models were fitted after controlling for age, sex, Hukou, marital status, education, pension insurance and annual household spending.*

* = p < 0.05

Child’s migration was found to have a significant association with poor self-rated health, not satisfied with general life and seeking outpatient visits among older parents with chronic diseases left behind. The results show that the migration of child has an adverse effect on both physical health, mental health and health care utilization of older parents with chronic diseases left behind, which means a comprehensive effect on their health status. On the other hand, there were no significant associations found between child’s migration and symptoms of physical limitations, symptoms of depression or inpatient visits of older parents with chronic diseases left behind.

Specifically, those parents with chronic diseases who had migrant child were more likely to report poor self-rated health (OR = 1.26; 95% CI 1.01–1.58), not satisfied with general life (OR = 1.28; 95% CI 1.03–1.59) and seek outpatient visits (OR = 1.22; 95% CI 1.03–1.43) than those who had no migrant child (Table 4).

## Discussion

This research attempted to explore the impact of child’s migration on health status and health care utilization of older parents with chronic diseases left behind in China, where general up to adult child to bear the responsibility for their older parents.

Results indicate that part of the older patients with chronic diseases had migrant child and migration of child was found negatively associated with both health status and health care utilization of older parents with chronic diseases left behind. Similar negative association was found in previous studies as well [22, 23].

For health status, specifically, after controlling for other demographic and socioeconomic variables, it was found that those who with migrant child were more likely to report poor self-rated health and not satisfied with general life than those who without migrant child, which was similar to the conclusion of other related studies [41, 42]. It is probably because that child’s migration reduces opportunities of face-to-face communication between parents and child, which increases the loneliness and isolation felt by their older parents left behind [21] and is difficult to address through formal care mechanisms such as hired help [43, 44]. These feelings of loneliness and isolation lead to persistent anxiety, worry and sadness of older parents [5], which may impair their physical and mental health in the long run. Besides, child’s intergenerational support is especially important when their aged parents become infirm [45], which is reflected clearly in this study of left-behind parents with chronic diseases. However, child’s migration status was not associated with symptoms of physical limitations or depression of their left-behind parents in this study. This may be due to the fact that adult child is less likely to migrate if their parents are in seriously bad condition [46], especially those whose parents have chronic diseases.

As to health care utilization, specifically, the older parents who with migrant child were more likely to seek outpatient visits than those whose child had not migrated after controlling for other demographic and socioeconomic variables. Similar results have also been found in other related studies that out-migration of adult child was associated with higher utilization of health services among older parents left behind [46]. One of the possible explanations for this finding is that left-behind parents suffering from chronic diseases lack healthcare from their migrant child, so they have to seek outpatient visits more frequently than those whose child live in closer proximity. Another possible explanation is remittances from migrant child which make outpatient visits affordable. The economic support provided by migrant child has been reported contributed positively to the material welfare of parents left behind [47], who tend to benefit economically [48] and to seek treatment for their diseases [46]. On the other hand, adult child’s migration status was not associated with the utilization of inpatient treatment among older parents with chronic diseases left behind. This may be due to the aforementioned reason that adult child is less likely to migrate when their parents are in bad health [46], especially those whose parents have chronic diseases.

Certain limitations should be taken into account when interpreting the findings of this study. Firstly, given that the study’s cross-sectional design, all factors analyzed in this study were measured at one point in time. Therefore, it is not possible for this study to ascertain the cause-effect relationship between adult child’s migration status and health outcomes of older parents with chronic diseases left behind. Analyses conducted in this study can only provide evidence of the statistical association between the two. Secondly, we must be cautious when comparing the findings of this study with those of other studies using other questionnaires to measure health outcomes.

## Conclusion

Despite its limitations, our study helps to solve the current issue of migration of adult child which has received little attention. Using a nationally representative sample of the aged, the results of this study show a statistically significant relationship between the migration of adult child and poor self-rated health, not satisfied with general life and seeking outpatient visits among older parents with chronic diseases left behind, which indicates a comprehensive effect on both their health status and health care utilization. Thus, in order to maintain and enhance the welfare of older parents with chronic diseases left behind, it will be important to balance economic growth and urbanization with the maintenance and enhancement of population health from a policy perspective. It is necessary to establish an effective community medical services and healthcare insurance system to guarantee the medical conditions and health outcomes of left-behind chronic diseases patients. Besides, policy set by the government should also aim to reduce the disparities in health care utilization among older parents who live with their child and older parents left behind. Communities can establish autonomous organizations among seniors with chronic diseases, which may increase their mutual assistance and reduce the health care utilization caused by their child’s migration.

## Data Availability

The datasets analyzed in this study are publicly available at the China Health and Retirement Longitudinal Study (CHARLS) site: http://charls.pku.edu.cn/en/page/data/2015-charls-wave4.

## Abbreviations

CHARLS: China Health and Retirement Longitudinal Study
WHO: World Health Organization
SRH: Self-rated health
ADL: Activities of daily living
IADL: Instrumental activities of daily living
SRLS: Self-rated life satisfaction
CES-D-10: 10-item Center for Epidemiologic Studies Depression Scale
